# Association between physical activity level and gut microbiota profiles in individuals with subthreshold depression: a cross-sectional study

**DOI:** 10.3389/fmicb.2026.1887394

**Published:** 2026-08-20

**Authors:** Meihua Su, Jiahui Jin, Fengxun Lin, Shihui Yang, YingYing Diao, Zange Lin

**Affiliations:** 1 Physical Education Institute, Jimei University, Xiamen, Fujian, China; 2 Psychological Counseling Center, Jimei University, Xiamen, Fujian, China

**Keywords:** cardiorespiratory fitness, cross-sectional study, gut microbiota, physical activity, subthreshold depression

## Abstract

**Background:**

Subthreshold depression (SD) represents a subclinical state between mental health and major depressive disorder (MDD) and is associated with a high prevalence and substantial risk of progression to MDD. Physical activity (PA) has been proposed to exert antidepressant effects through modulation of the gut–brain axis. However, the association between PA level and gut microbiota profiles in individuals with SD remains poorly understood.

**Methods:**

This is a cross-sectional observational study. Stratified random sampling was used to screen full-time undergraduates aged 18–25 years at Jimei University for subthreshold depression. Following initial screening with the CES-D and secondary screening with a structured clinical interview, the HAMD-17, and the BDI-II, 122 participants were enrolled. Participants were assigned by IPAQ short form to low (*n* = 46), moderate (*n* = 47), and high (*n* = 29) physical activity groups. Cardiorespiratory fitness was assessed as maximal oxygen uptake estimated by a submaximal Astrand-Rhyming cycle ergometer test. Fecal samples were subjected to 16S rRNA gene V3–V4 sequencing; α-diversity, β-diversity, differential genera, and keystone species were analyzed.

**Results:**

No significant differences in α-diversity or β-diversity were observed among the three PA groups (*P* > 0.05). However, distinct taxonomic signatures were identified. The low-PA group was enriched in potentially pro-inflammatory taxa, including *Streptococcaceae* and *Eggerthella* (*P* < 0.05), whereas the moderate-PA group exhibited increased abundance of butyrate-producing genera, including *Roseburia* and *Lachnospiraceae_UCG-010* (*P* < 0.05). The high-PA group showed significant enrichment of Dorea (*P* < 0.05). *Faecalibacterium* exhibited the highest Structural Keystoneness (Ks) within the microbial co-occurrence network, and its relative abundance was positively associated with VO_2_max. Moreover, Median (Ks) was strongly correlated with MAD (Ks) (ρ = 0.602, *P* < 0.001), indicating that the ecological importance of high-keystone taxa is highly context dependent.

**Conclusion:**

Although PA level was not significantly associated with overall gut microbial diversity in college students with SD, it was closely associated with the differential distribution of specific functional genera. These findings provide novel evidence supporting the role of PA-related microbial remodeling in SD and may facilitate the development of microbiota-targeted interventions that mimic exercise-induced benefits.

## 1 Introduction

Subthreshold depression (SD) is a subclinical condition characterized by clinically relevant depressive symptoms that do not fully meet the diagnostic criteria for major depressive disorder (MDD) (Rodríguez et al., 2012). Despite its subclinical nature, SD is highly prevalent and is associated with substantial psychosocial impairment and an elevated risk of progression to MDD. Epidemiological studies have reported a lifetime prevalence of approximately 11% in the general population, with nearly 20%–30% of affected individuals subsequently developing MDD within 1 year (Zhang et al., 2023). In addition, individuals with SD often exhibit social dysfunction and reduced quality of life comparable to those observed in mild-to-moderate MDD (Jinnin et al., 2017). Early intervention for SD may effectively reduce the risk of future depressive episodes and alleviate symptom burden (Cuijpers et al., 2014); Although psychotherapy remains the primary recommended treatment strategy, its clinical implementation is often limited by high cost, prolonged treatment duration, variable treatment response, and poor adherence (Krishna et al., 2015). Consequently, increasing attention has been directed toward safe, accessible, and non-pharmacological interventions, particularly exercise-based approaches.

Accumulating evidence supports the beneficial effects of physical activity (PA) on emotional health (Noetel et al., 2024). A recent meta-analysis demonstrated that regular PA reduces the risk of depression by approximately 18%–25%, with moderate- and vigorous-intensity PA showing greater protective effects (Pearce et al., 2022). Emerging evidence further suggests that the antidepressant effects of PA may be partially mediated through the gut–brain axis (Makin, 2025). Animal studies have demonstrated that voluntary wheel running increases the abundance of short-chain fatty acid (SCFA)-producing bacteria and alleviates anxiety-like behaviors (Yao et al., 2025). Similarly, athletes exhibit greater gut microbial diversity and distinct microbial compositions compared with sedentary individuals (Clark and Mach, 2016). Emerging evidence suggests that the gut–brain axis constitutes an important biological pathway linking physical activity and emotional health. Exercise-induced alterations in gut microbiota composition and microbial metabolites, particularly short-chain fatty acids, may modulate neuroinflammation, neurotransmitter metabolism, and intestinal barrier function, thereby influencing depressive symptoms (Góralczyk-Bińkowska et al., 2022). Collectively, these findings suggest that the gut microbiota may represent a key biological mediator linking PA to emotional regulation.

Although physical activity exerts antidepressant effects through multiple mechanisms, including enhanced neuroplasticity, regulation of monoamine neurotransmitters, improved hypothalamic-pituitary-adrenal (HPA) axis function, and reduced systemic inflammation (Sutherland, 2019), the gut–brain axis has emerged as a particularly attractive target because of its potential translational value (Patel et al., 2025). In clinical practice, a substantial proportion of individuals with depressive symptoms fail to adhere to long-term exercise interventions due to low motivation, physical limitations, or exercise intolerance. Therefore, identifying gut microbial signatures associated with physical activity may facilitate the development of microbiota-targeted strategies, such as probiotics, prebiotics, and microbial metabolite-based therapies, which may partially mimic exercise-induced benefits and provide alternative interventions for individuals unable to engage in regular exercise.

Gut microbial dysbiosis has been increasingly implicated in depression. Patients with MDD commonly exhibit reduced microbial diversity, depletion of butyrate-producing bacteria, and enrichment of pro-inflammatory taxa (Liu et al., 2023). These alterations may contribute to depressive pathophysiology through multiple mechanisms, including disruption of intestinal barrier integrity, chronic low-grade inflammation, altered tryptophan metabolism, and impaired vagal signaling (Liu et al., 2024). However, studies investigating gut microbiota characteristics during the SD stage remain limited. Individuals with SD may already exhibit early microbial dysbiosis resembling that observed in MDD, although to a lesser extent. To date, no study has systematically explored the relationship between PA level and gut microbiota profiles in individuals with SD.

College students constitute a particularly vulnerable population for subthreshold depression due to high levels of academic pressure, psychosocial stress, and lifestyle transitions. Because this developmental stage provides a critical window for early intervention, investigating microbiota-related mechanisms in college students may offer valuable insights for preventing progression to major depressive disorder.

Therefore, the present study aimed to investigate differences in gut microbial composition, diversity, and keystone taxa across varying PA levels in medication-free college students with SD. Participants were stratified into low-, moderate-, and high-PA groups according to IPAQ-SF scores. our study aims to provide preliminary evidence regarding microbiota-related mechanisms underlying exercise and to identify potential targets for early preventive interventions

## 2 Methods

### 2.1 Study design

This cross-sectional observational study was conducted at Jimei University and reported in accordance with the Strengthening the Reporting of Observational Studies in Epidemiology (STROBE) guidelines. The study protocol was approved by the Academic Ethics Committee of Jimei University (Approval No. JMU202501001). Written informed consent was obtained from all participants prior to enrollment.

Participants were recruited between December 2024 and June 2025 using a stratified random sampling strategy. Stratification was performed according to academic year, and students were randomly selected from the university registration database. Eligible participants subsequently underwent a two-stage screening procedure for SD.

Following enrollment, all participants completed a 3-day dietary standardization protocol before fecal sample collection. Physical activity assessment, cardiorespiratory fitness testing, demographic data collection, and fecal sampling were conducted during the same assessment period.

### 2.2 Screening procedure and diagnostic criteria

The screening procedure consisted of online preliminary screening followed by in-person clinical assessment.

During preliminary screening, participants completed the Center for Epidemiologic Studies Depression Scale (CES-D) and Symptom Checklist-90 (SCL-90). Individuals meeting the following criteria were eligible for secondary screening: CES-D score ≥16, age 18–25 years, and full-time undergraduate status.

Participants were excluded if they demonstrated severe psychiatric symptoms, suicidal tendencies, current psychotropic medication use, gastrointestinal disease, recent antibiotic or probiotic exposure, or contraindications to exercise testing.

Secondary screening included structured clinical interviews, the Hamilton Depression Rating Scale-17 (HAMD-17), and the Beck Depression Inventory-II (BDI-II). SD was diagnosed according to MINI version 5.0 and established operational criteria.

### 2.3 Physical activity assessment

PA was assessed using the International Physical Activity Questionnaire Short Form (IPAQ-SF). Trained investigators conducted face-to-face interviews to record the frequency and duration of walking, moderate-intensity PA, and vigorous-intensity PA during the preceding 7 days.

Participants were categorized into low- (PA_Low), moderate- (PA_Medium), and high-PA (PA_High) groups according to IPAQ scoring guidelines. Daily sedentary time was also recorded.

### 2.4 Cardiorespiratory fitness assessment

Maximal oxygen uptake (VO^2^max) represents the gold standard for assessing cardiorespiratory fitness. Both direct and indirect methods are commonly employed to measure VO^2^max, with a reported correlation coefficient of 0.87 between the two approaches (Warburton et al., 2004).

Cardiorespiratory fitness was evaluated using estimated maximal oxygen uptake (VO^2^max) obtained from a submaximal Åstrand–Ryhming cycle ergometer test. Participants performed cycling exercise at a constant cadence of 60 rpm while wearing a heart rate monitor.

Relative VO^2^max was estimated using workload, body weight, and steady-state heart rate values obtained during the fifth and sixth minutes of exercise. Participants were instructed to avoid vigorous exercise, alcohol, and caffeine for 24 hours before testing.

### 2.5 Fecal sample collection and 16S rRNA sequencing

Fresh fecal samples were collected on the morning following completion of dietary standardization. Samples were immediately transported to the laboratory under cold-chain conditions and stored at -80 °C until analysis.

Total genomic DNA was extracted using the PowerSoil Pro Kit. The V3–V4 regions of the bacterial 16S rRNA gene were amplified using universal primers (341F/806R). Sequencing libraries were constructed and sequenced on the Illumina NovaSeq platform. Refer to Supplementary Method S1 for details.

### 2.6 Sample size estimation

The Shannon index of gut microbial α-diversity was prespecified as the primary outcome measure. Because no previous studies have reported differences in gut microbial α-diversity across physical activity levels in individuals with subthreshold depression, assumptions regarding the effect size were informed by analogous studies examining associations between physical activity and gut microbiota composition in healthy adults. Estaki et al. (2016) reported an approximately 0.5-unit difference in the Shannon index between individuals with higher and lower cardiorespiratory fitness, independent of self-reported physical activity. Based on these findings and Cohen’s recommendations for ANOVA effect sizes, a conservative medium effect size (f = 0.30) was adopted for the a priori power analysis. Sample size estimation was performed using G*Power software (version 3.1.9.7) for a one-way ANOVA (fixed effects, omnibus test) with three groups, a two-sided significance level of α = 0.05, and a statistical power of 80% (1 - β = 0.80). The minimum required sample size was estimated to be 111 participants. Considering a potential attrition rate of approximately 10% due to failed fecal DNA extraction, missing questionnaire data, or incomplete dietary records, we planned to recruit at least 122 participants with subthreshold depression.

### 2.7 Quality control

To minimize dietary confounding before fecal sample collection, Participants completed a 3-day dietary survey prior to fecal sample collection to characterize their habitual dietary intake. No identical meals or nutritionally standardized diets were provided, and participants were not instructed to modify their usual eating habits during this period. Dietary intake was assessed using 3-day dietary records, which were reviewed by trained investigators for completeness and compliance. Consumption of probiotic products, fermented foods (e.g., yogurt, kimchi), alcohol, dietary supplements, and spicy foods was prohibited. Participants were instructed not to consume meals outside the designated cafeteria except for plain water. Compliance was monitored using real-time electronic dietary records with meal photographs uploaded after each meal, and all records were reviewed daily by a registered dietitian. Daily energy intake, macronutrient composition, and dietary fiber intake were calculated using the China Food Composition Table. Details of the 3-day dietary assessment procedure and the dietary record form are provided in Supplementary Method S2. All study personnel involved in testing procedures underwent standardized training and passed qualification assessments before performing study-related tasks; all data were independently entered and verified in duplicate by two investigators to prevent data entry errors, and all statistical analyses were performed by a third investigator.

### 2.8 Statistical analysis

Statistical analyses were performed using SPSS version 27.0 (IBM Corp., Armonk, NY, USA) and GraphPad Prism version 8.0 (GraphPad Software, San Diego, CA, USA). Continuous variables are presented as mean ± standard deviation (SD) or median (interquartile range, IQR), depending on data distribution. Raw sequencing data were processed using the QIIME2 pipeline, and amplicon sequence variants (ASVs) were generated using the DADA2 denoising algorithm. Between-group differences in continuous variables were assessed using one-way analysis of variance (ANOVA) or the Kruskal–Wallis test, as appropriate. Pearson or Spearman correlation analyses were performed according to data distribution. For taxon-level differential abundance analyses, multiple-testing correction was performed using the Benjamini–Hochberg false discovery rate (FDR) procedure, and the corresponding adjusted q values were reported. Taxa with an adjusted *q* value < 0.05 were considered statistically significant. All statistical tests were two-sided, and *P* < 0.05 (or adjusted *q* < 0.05, where applicable) was considered statistically significant.

## 3 Results

### 3.1 Participant characteristics

Analysis of the 122 valid samples revealed the following: 59 males and 63 females, with a mean age of 20 years; the PA_Low group comprised 46 participants (37.70%), the PA_Medium group 47 (38.52%), and the PA_High group 29 (23.77%). According to the physical activity questionnaire, the PA_Low group predominantly engaged in sedentary behavior or minimal walking; the PA_Medium group primarily undertook regular, sustained walking; and the PA_High group mainly participated in cycling and jogging. One-way ANOVA revealed no statistically significant differences among the PA_Low, PA_Medium, and PA_High groups in age, height, body weight, BMI, or dietary factors (*P* > 0.05); however, maximal oxygen uptake differed significantly with increasing physical activity level (*P* < 0.01) (Table 1).

**TABLE 1:** Baseline characteristics of participants.

	Physical activity level grouping			*F*	*p*
	PA_Low (*n* = 46)	PA_Medium (*n* = 47)	PA_High (*n* = 29)	*F*-value	*P*-value
Sex n (%)					
Male	24 (52.17%)	21 (44.68%)	14 (48.27%)	0.256	0.775
Female	22 (47.82%)	26 (55.31%)	15 (51.72%)		
Age (y)	20.91 ± 2.00	20.68 ± 2.10	21.10 ± 2.11	0.393	0.676
Height (cm)	167.92 ± 10.62	167.66 ± 9.97	168.09 ± 11.94	0.016	0.984
Weight (kg)	69.44 ± 10.39	67.68 ± 13.64	68.80 ± 11.33	0.257	0.774
BMI (kg/m^2^)	24.62 ± 2.91	23.89 ± 3.15	24.32 ± 2.87	0.682	0.508
Maximal oxygen uptake (absolute)	2.26 ± 0.52	2.24 ± 0.53	3.23 ± 0.91	26.097	0.01**
Maximal oxygen uptake (relative)	32.65 ± 4.43	35.32 ± 5.96	47.03 ± 9.87	44.478	0.01**
BDI	8.24 ± 2.36	8.23 ± 2.49	8.14 ± 2.25	0.019	0.981
HAMD	10.83 ± 2.43	11.57 ± 3.11	11.41 ± 2.85	0.888	0.414
Carbohydrates intake (g/d)	249.26 ± 32.38	240.23 ± 38.22	256.01 ± 43.89	1.671	0.192
Protein intake (g/d)	64.53 ± 10.52	68.75 ± 9.44	64.68 ± 10.02	2.512	0.085
Fat intake (g/d)	35.47 ± 8.79	35.46 ± 8.20	34.21 ± 8.94	0.233	0.792
Dietary fiber (g/day)	18.6 ± 5.2	19.8 ± 5.5	20.3 ± 5.7	0.73	0.431
Total energy intake (kcal/d)	1574.35 ± 144.27	1555.02 ± 167.48	1590.65 ± 178.53	0.452	0.637
Physical activity (MET-h/d)	470.59 ± 79.71	1118.74 ± 206.44	1766.78 ± 150.51	630.164	0.01**

*P < 0.05, **P < 0.01.

### 3.2 Effect of physical activity level on gut microbial diversity

**FIGURE 1:**
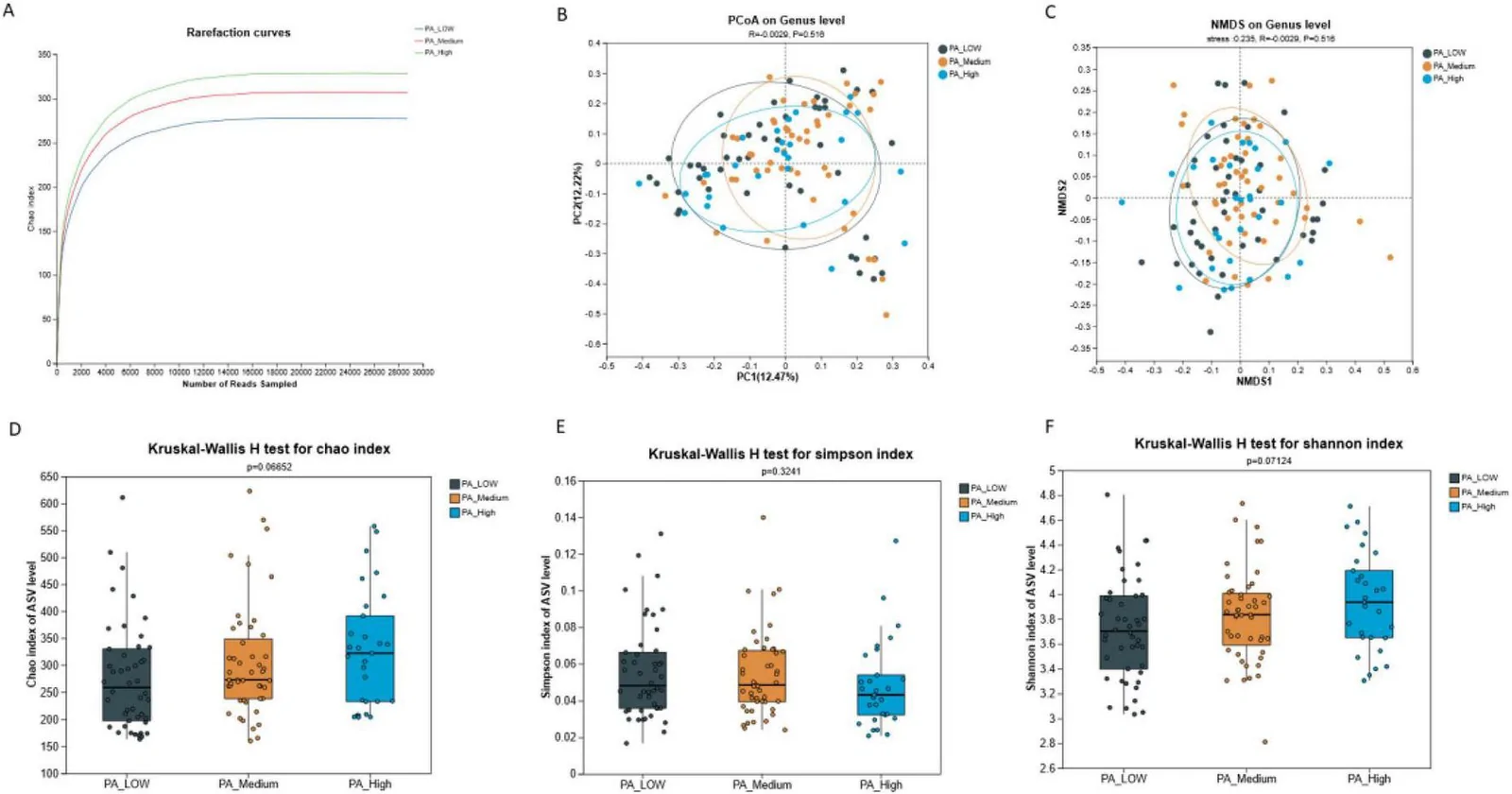
Effect of physical activity level on gut microbial diversity. **(A)** Rarefaction curves and Chao index box plots showing sequencing depth and estimated species richness for each group. **(B)** Principal coordinate analysis (PCoA) ordination based on genus-level Bray-Curtis distance. **(C)** Non-metric multidimensional scaling (NMDS) ordination based on genus-level community composition. **(D–F)** Intergroup comparisons of α-diversity metrics.

Rarefaction curve analysis revealed that all three physical activity groups approached a plateau, indicating that the sequencing depth was sufficient to capture the vast majority of microbial species in the samples and that the data volume met the requirements for subsequent analyses (Figure 1A); β-diversity analysis based on genus-level PCoA and NMDS revealed no significant overall structural differences among the three groups, although partial separation was observed (Figures 1B,C); these results suggest that the overall microbial community structure was not markedly distinct among subthreshold depression college students with different physical activity levels. For α-diversity, the Chao, Simpson, and Shannon indices showed no significant intergroup differences; although the Shannon and Chao indices did not reach statistical significance, discernible trends were observed (Figures 1D–F).

### 3.3 Effect of physical activity level on gut microbial composition

**FIGURE 2:**
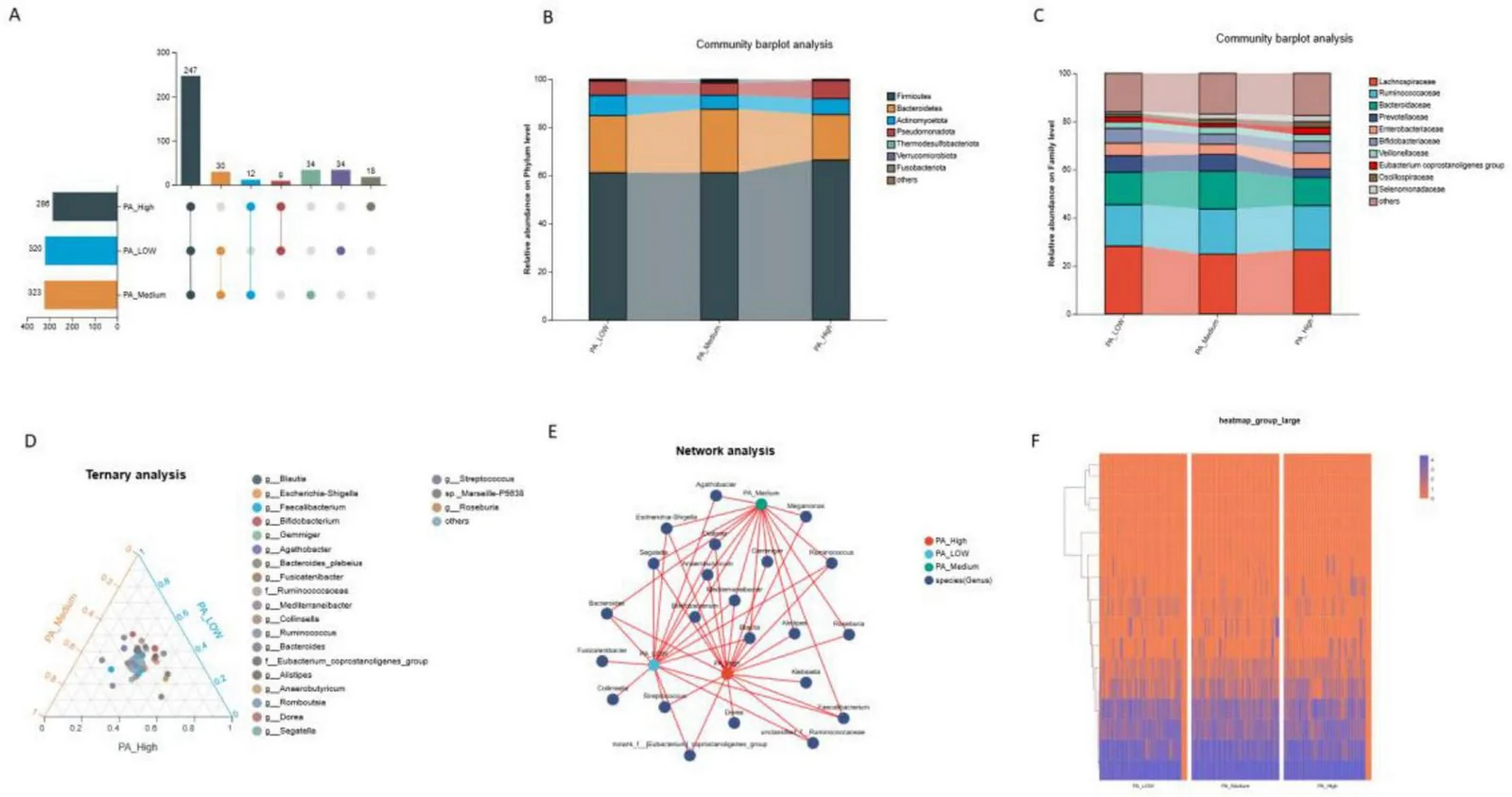
Effect of physical activity level on gut microbial composition. **(A)** UpSet plot comparing the distribution of shared and unique ASVs across groups. **(B)** Bar plot of community relative abundance at the phylum level. **(C)** Bar plot of community relative abundance at the family level. **(D)** Ternary plot showing the enrichment patterns of differential genera across physical activity levels. **(E)** Co-occurrence network analysis displaying the association patterns between physical activity groups and key genera. Red nodes represent the PA_High group, blue nodes the PA_Low group, green nodes the PA_Medium group, and gray nodes individual genera. Edge thickness reflects association strength. **(F)** Genus-level heatmap showing the relative abundance distribution of dominant genera and sample clustering across the three groups.

An UpSet plot was used to visualize the shared and unique amplicon sequence variants (ASVs) across the three groups. A core set of 247 ASVs was common to all three groups; the numbers of unique ASVs were 286, 320, and 323 in the PA_High, PA_Low, and PA_Medium groups, respectively (Figure 2A). At the phylum level, *Firmicutes* and *Bacteroidetes* dominated across all groups. The relative abundance of *Bacteroidetes* was slightly higher in the PA_Low group than in the moderate- and high-activity groups, with a corresponding decrease in the proportion of *Firmicutes* (Figure 2B). At the family level, *Lachnospiraceae*, *Ruminococcaceae*, and *Bacteroidaceae* were the dominant taxa across all groups, with their relative abundances showing distinct trends across physical activity levels (Figure 2C). To further delineate genus-level distribution patterns, ternary plot analysis was performed, revealing a tendency toward separation in the enrichment profiles of various genera among the three groups (Figure 2D). Network analysis further elucidated that the PA_High group displayed strong associations with SCFA producing genera such as *Faecalibacterium*, *Bifidobacterium*, *Roseburia*, and *Agathobacter*, suggesting that these beneficial taxa occupy a more central ecological niche in this group. In contrast, the PA_Low group was more closely associated with *Escherichia-Shigella*, *Alistipes*, and *Klebsiella*; notably, *Escherichia-Shigella* and *Klebsiella* are members of Enterobacteriaceae and represent potentially pro-inflammatory opportunistic pathogens. The PA_Medium group exhibited robust co-occurrence relationships with *Blautia*, *Mediterraneibacter*, *Collinsella*, and *Dorea*, displaying a transitional microbial association profile intermediate between the high- and low-activity groups (Figures 2E, F).

### 3.4 Comparison of differential taxa across physical activity levels

**FIGURE 3:**
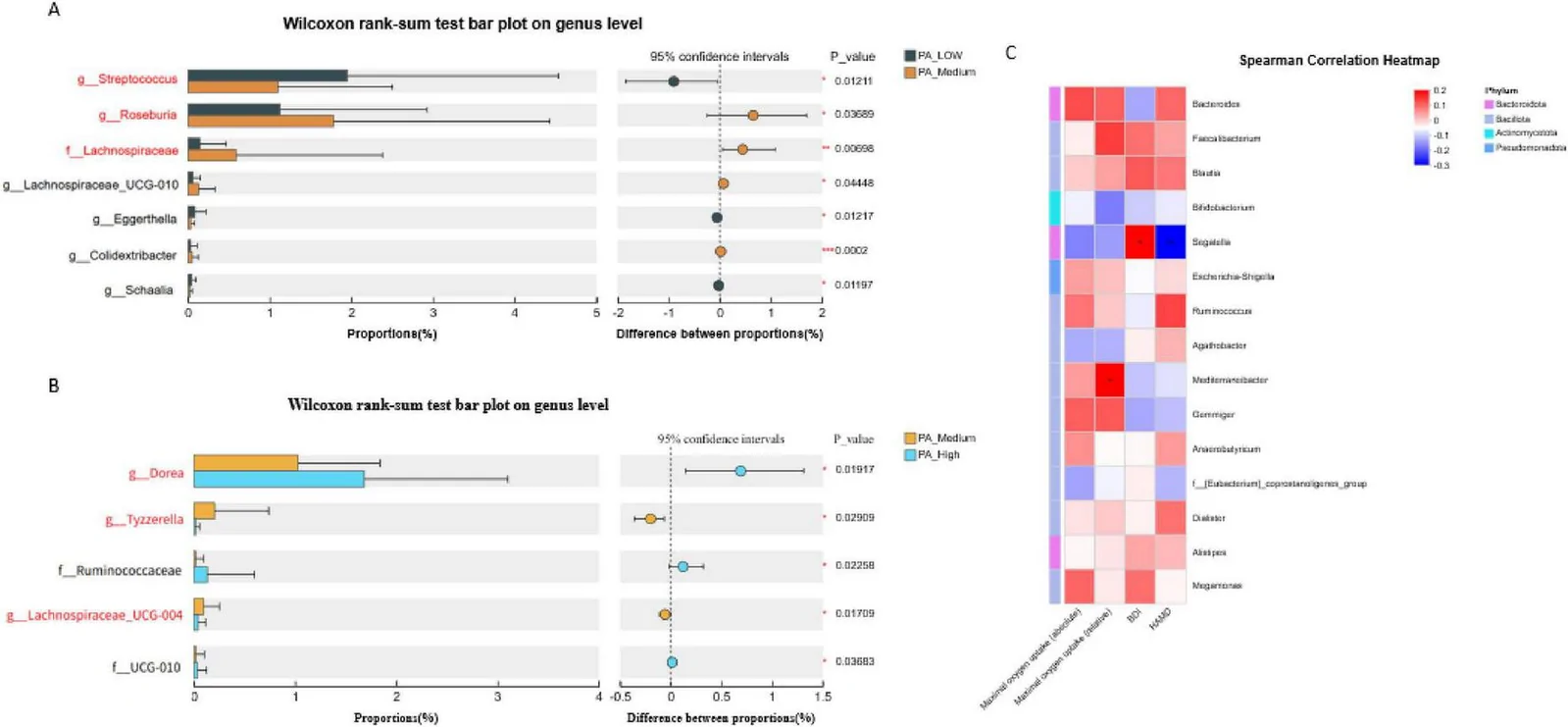
Comparison of Differential Taxa Across Physical Activity Levels. **(A)** Genus-level differential taxa between the PA_Medium and PA_High groups. **(B)** Genus-level differential taxa between the PA_Low and PA_Medium groups. **(C)** Spearman correlation heatmap showing associations between genus-level abundances and clinical phenotypes.

To further delineate the distribution patterns of differential genera across physical activity levels, pairwise comparisons among the three groups were performed using the Wilcoxon rank-sum test, followed by Benjamini–Hochberg false discovery rate (FDR) correction for multiple comparisons. Comparison between the PA_Low and PA_Medium groups identified seven genera that remained significantly different after FDR correction (*q* < 0.05). Specifically, Streptococcaceae (*P* = 0.0018, *q* = 0.013) and Eggerthella (*P* = 0.0065, *q* = 0.027) were significantly enriched in the PA_Low group, whereas Roseburia (*P* = 0.0023, *q* = 0.015) and Lachnospiraceae_UCG-010 (*P* = 0.0089, *q* = 0.034) were significantly enriched in the PA_Medium group (Figure 3A, Supplementary Figures 1A–C). Notably, the enrichment of the butyrate-producing genus Roseburia in the PA_Medium group suggests that moderate levels of physical activity may be associated with a more favorable gut microbial profile. Comparison between the PA_Medium and PA_High groups identified five genera that remained significantly different after FDR correction (*q* < 0.05). Dorea (*P* = 0.0041, *q* = 0.021) was significantly enriched in the PA_High group, whereas Tyzzerella (*P* = 0.0078, *q* = 0.032) and Lachnospiraceae_UCG-004 (*P* = 0.0096, *q* = 0.038) showed significantly higher relative abundance in the PA_Medium group (Figure 3B, Supplementary Figures 1D–F). Relative VO^2^max was positively correlated with the relative abundance of *Bacteroides* (*P* < 0.05); absolute VO^2^max was positively correlated with *Mediterraneibacter* and *Faecalibacterium* (*P* < 0.05); *Segatella* relative abundance was negatively correlated with HAMD scores (*P* < 0.01). Additionally, *Blautia* and *Gemmiger* displayed positive correlation trends with maximal oxygen uptake, and *Bifidobacterium* showed negative correlation trends with both HAMD and BDI scores, although none reached statistical significance (Figure 3C). Notably, *Faecalibacterium*, which was positively correlated with cardiorespiratory fitness, also showed an enrichment trend in the high-activity group, whereas *Bifidobacterium*, which was negatively correlated with HAMD and BDI scores, tended to be enriched in the low-activity group (Figures 2D, E).

### 3.5 Effect of physical activity level on keystone taxa

**FIGURE 4:**
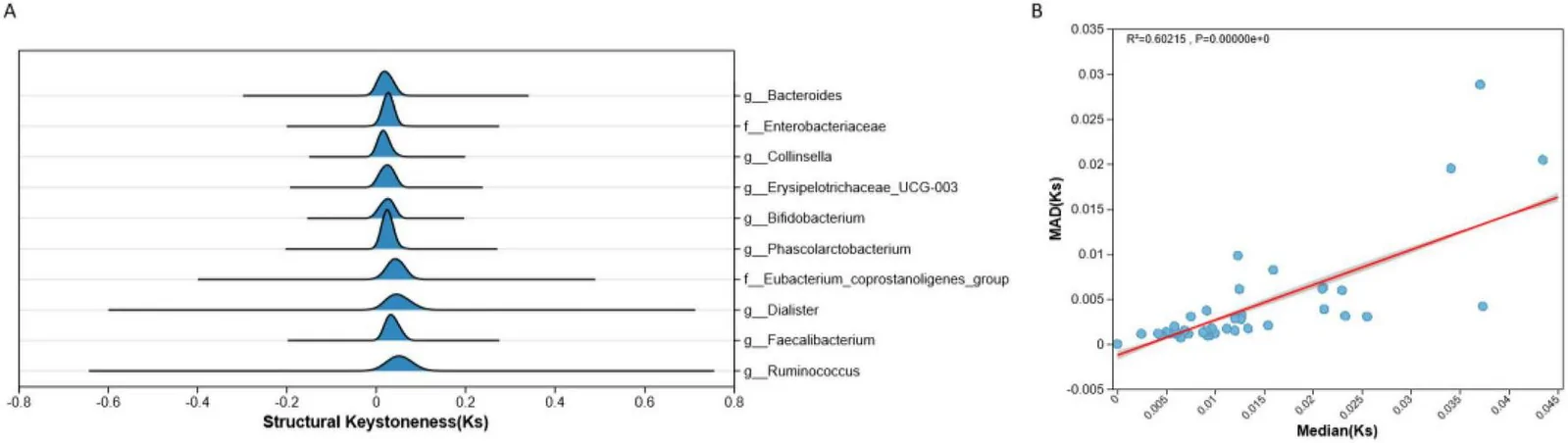
Effect of physical activity level on keystone taxa. **(A)** Distribution of Structural Keystoneness (Ks) scores across all detected genera. **(B)** Spearman correlation between median keystone-ness [median (Ks)] and the median absolute deviation of keystone-ness [MAD (Ks)].

As shown in Figure 4A and Supplementary Table 1, *Faecalibacterium* exhibited the highest Structural Keystoneness (Ks) score, indicating that this genus occupies the most central ecological niche within the entire microbial co-occurrence network; even subtle fluctuations in its relative abundance could substantially impact the overall stability of the community network. *Ruminococcus* and *Dialister* ranked next, both displaying relatively high Ks values, suggesting that they play important supporting roles in maintaining the network architecture. To systematically investigate the community specificity of these keystone taxa, we plotted median keystone-ness [median (Ks)] against the median absolute deviation of keystone-ness across all samples [MAD (Ks)]. This analysis revealed a significant correlation between MAD (Ks) and median (Ks), with a Spearman correlation coefficient ρ = 0.602 (Figure 4B).

## 4 Discussion

In this study, we compared the composition, diversity, and keystone taxa characteristics of the gut microbiota across low, moderate, and high physical activity levels in medication-free college students with subthreshold depression. Our principal findings are threefold. First, no significant differences in overall α- or β-diversity were detected among the three groups, suggesting that physical activity level does not substantially alter overall species richness or community structure, yet significant differences in taxonomic composition were evident. Second, specific functional genera displayed distinct distribution patterns across activity levels: the low-activity group was characterized by enrichment of pro-inflammatory taxa such as *Streptococcaceae* and *Eggerthella*; Moderate physical activity was associated with the enrichment of butyrate-producing bacteria, including Roseburia and Lachnospiraceae_UCG-010. Because these taxa have been linked to intestinal barrier maintenance and anti-inflammatory processes in previous studies, their increased abundance may indicate a potentially favorable microbial profile; and the high-activity group was enriched for beneficial genera such as *Dorea*. Third, keystone species analysis revealed that *Faecalibacterium* occupied the most central position within the microbial co-occurrence network. Collectively, these findings indicate that the impact of physical activity on the gut microecology of college students with subthreshold depression manifests not as an increase in overall microbial diversity, but rather as a targeted regulation of specific functional genera and their ecological network positions.

The absence of significant differences in α- and β-diversity across physical activity levels may be attributable to variations in exercise intensity and duration (Dorelli et al., 2021); in contrast, athletes undergoing long-term, regular high-intensity training typically exhibit significantly higher α-diversity than their sedentary counterparts (Jung et al., 2020). Thus, although individuals who regularly engage in physical activity accrue multifaceted health benefits, changes in overall gut microbial diversity may remain inconspicuous when activity levels fall below those characteristic of athletic training. Notably, despite the lack of statistical differences in microbial diversity, marked alterations in taxonomic composition and community structure were observed. Wang et al. (2023) similarly reported that 12 weeks of moderate aerobic exercise did not significantly alter α- or β-diversity in adolescents, yet substantial remodeling of gut microbiota composition and structure occurred. Moreover, elevated α-diversity does not invariably equate to improved gut health and may, under certain circumstances, reflect the overgrowth of potentially harmful microorganisms. Consequently, in studies examining the association between physical activity and the gut microbiota, a focus on compositional shifts and differential taxa may carry greater biological significance than an exclusive emphasis on α-diversity. Importantly, increased microbial diversity should not automatically be interpreted as indicative of improved gut health. Greater diversity may also result from the expansion of opportunistic pathogens or functionally redundant taxa (Lozupone et al., 2012). Recent ecological studies have emphasized that microbial functions, network organization, and the abundance of specific keystone taxa may provide more biologically meaningful information than diversity indices alone (Hooks and O’Malley, 2017). Therefore, the absence of significant differences in α- and β-diversity among physical activity groups does not exclude the possibility of exercise-induced microbial remodeling. Instead, our findings suggest that physical activity preferentially reshapes the abundance and ecological roles of specific functional taxa, particularly SCFA-producing and inflammation-related bacteria.

In the present medication-free cohort, we found that the low-activity group was enriched for potentially pro-inflammatory taxa such as *Streptococcaceae* and *Eggerthella*: *Streptococcaceae* may promote the release of pro-inflammatory cytokines including IL-6 and TNF-α through TLR-mediated NF-κB signaling, thereby contributing to neuroinflammatory processes and exacerbating depressive symptoms (Tang et al., 2025), while *Eggerthella* has been implicated in the induction of peripheral inflammation that may predispose individuals to depression (Edwin Thanarajah et al., 2025). The moderate-activity group was enriched for *Roseburia*, a major colonic butyrate producer whose metabolite butyrate serves not only as an energy substrate for colonocytes but also exerts anti-inflammatory and gut barrier-protective effects through histone deacetylase inhibition and G protein-coupled receptor activation (Zhao et al., 2024). The high-activity group was enriched for *Dorea*, a member of the *Lachnospiracea*e within the *Firmicutes* phylum, which possesses broad metabolic capabilities including SCFA synthesis, mucin degradation, and sugar and aromatic amino acid metabolism (Palmnäs-Bédard et al., 2022); although *Dorea* has been classified as pro-inflammatory in some studies, more recent evidence demonstrates its reduced abundance in patients with depression and a negative correlation with depression severity (Yu et al., 2024), and a randomized controlled trial in adolescents with subthreshold depression further showed that 3 months of moderate-intensity exercise significantly restored *Dorea* abundance (Estaki et al., 2016). In the co-occurrence network analysis, *Faecalibacterium* occupied the most central structural position; as a key butyrate producer, this genus exerts anti-inflammatory effects, maintains bacterial enzyme activity, and protects the digestive system from enteric pathogens (Lopez-Siles et al., 2017). Ramos et al. (2025) reported that the relative abundance of SCFA-producing genera was significantly higher in regularly active individuals than in sedentary subjects, with a correspondingly lower abundance of pro-inflammatory Proteobacteria. Notably, VO^2^max was positively associated with the relative abundance of Faecalibacterium, which exhibited the highest Structural Keystoneness (Ks) in the microbial network. As an important butyrate-producing genus, Faecalibacterium has been linked to anti-inflammatory effects and cardiometabolic health (Dorelli et al., 2021). These findings suggest that cardiorespiratory fitness may represent an important component of the physical activity–gut microbiota–mental health axis. However, given the cross-sectional design, these associations should be interpreted cautiously and require confirmation in future longitudinal studies. Nevertheless, the antidepressant effects of exercise are likely multifactorial and cannot be solely attributed to gut microbiota modulation. Exercise has been shown to enhance neuroplasticity, increase brain-derived neurotrophic factor (BDNF) expression, regulate monoaminergic neurotransmission, improve HPA-axis function, and reduce systemic inflammation (Makin, 2025). Gut microbiota remodeling should be considered one potential mechanism within a broader network of exercise-induced physiological adaptations.

Thus, the promoting effect of high physical activity on *Faecalibacterium* may enhance butyrate synthesis, thereby directly improving gut barrier integrity and anti-inflammatory status, while strengthening the resilience of the entire gut microbial ecosystem against disease-related perturbations.

## 5 Conclusion

In medication-free college students with SD, PA level was not significantly associated with overall gut microbial diversity but was closely associated with the distribution of specific functional genera. Low PA was characterized by enrichment of potentially pro-inflammatory taxa, whereas moderate and high PA were associated with increased abundance of beneficial SCFA-producing bacteria.

*Faecalibacterium* demonstrated the highest ecological centrality within the microbial network and was positively associated with cardiorespiratory fitness, suggesting that this genus may represent an important mechanistic link within the PA–gut microbiota–mood regulation axis.

These findings provide novel evidence supporting exercise-associated microbial remodeling in SD and may contribute to the development of microbiota-targeted therapeutic strategies.

Collectively, our findings suggest that moderate-intensity physical activity, rather than simply pursuing higher exercise intensity, may be associated with a more favorable gut microbial profile linked to mental health in individuals with subthreshold depression.

## 6 Limitations

Several limitations should be acknowledged. First, the cross-sectional design precludes causal inference. Longitudinal and interventional studies are required to establish causal relationships between PA and gut microbiota alterations.

Second, PA was assessed using self-reported questionnaires, which may introduce recall bias and activity misclassification. Third, VO^2^max was estimated indirectly rather than measured using gas exchange analysis.

Finally, 16S rRNA sequencing provides limited taxonomic and functional resolution compared with metagenomic sequencing. A further limitation is that no standardized meals were provided before fecal sample collection. Although habitual dietary intake was assessed using a 3-day dietary record and no significant differences in energy, macronutrient, or dietary fiber intake were observed among groups, residual dietary variation cannot be completely excluded and may have influenced gut microbial composition. Future studies using controlled feeding protocols are warranted.

## Data Availability

The raw sequence data reported in this paper have been deposited in the Genome Sequence Archive (Genomics, Proteomics & Bioinformatics 2025) in National Genomics Data Center (Nucleic Acids Res 2025), China National Center for Bioinformation / Beijing Institute of Genomics, Chinese Academy of Sciences (GSA: CRA047423) that are publicly accessible at https://ngdc.cncb.ac.cn/gsa.
