# Supplementary material for: Association between physical activity level and gut microbiota profiles in individuals with subthreshold depression: a cross-sectional study
Source: Front Microbiol. 2026 Aug 20;17:1887394. doi: 10.3389/fmicb.2026.1887394 (PMC13538118; doi:10.3389/fmicb.2026.1887394)
Supplement: Supplementary file 1 [file Supplementary_file_1.docx]

Supplementary Material

**Association Between Physical activity level and Gut Microbiota Profiles in Individuals with Subthreshold Depression: A Cross-Sectional Stud**

Meihua Su 1†, Jiahui Jin1†, Fengxun Lin1, Shihui Yang1, YingYing Diao1, Zange Lin2

1 Physical Education Institute, Jimei University, xiamen, fujian, 361021, China

2 Psychological Counseling Center, Jimei University, xiamen, fujian, 361021, China

†:These authors contributed equally to this work

Corresponding author: Zange Lin

Telephone number: +86 (0592)6180035

Email: zangelin@jmu.edu.cn

**SUPPLEMENTARY MATERIAL**

**Supplementary Methods:**

**Supplementary method 1:** Stool sampling, fecal microbial DNA extraction,

amplification, and 16S rRNA gene sequencing

**Supplementary method 2:** Three-Day Dietary Assessment

**Supplementary Tables:**

**Supplementary Tables1:** Complete list of Ks value rankings for each genus in species key analysiss

**Supplementary Figures:**

**Supplementary Figures1:** Comparison of the relative abundance of different species

**Supplementary method 1:** Stool sampling, fecal microbial DNA extraction,

amplification, and 16S rRNA gene sequencing

(1) Fecal sample collection and storage

Fecal samples were collected from all participants at the designated time point. Participants were instructed to wash their hands and void urine prior to sample collection. Fresh fecal samples were obtained from the first spontaneous bowel movement in the morning under fasting conditions. To minimize environmental contamination, approximately 10 ± 5 g of feces was collected from the central portion of the stool (avoiding surface exposure) and placed into sterile collection tubes. Samples were labeled with participant identifiers and group codes, sealed, and immediately transported to a −80 °C freezer for storage until further analysis.

(2) DNA extraction and PCR amplification

Total genomic DNA was extracted from fecal samples using the cetyltrimethylammonium bromide (CTAB) method, followed by purification. DNA quality and concentration were assessed by 1% agarose gel electrophoresis, and samples were diluted to 1 ng/μL. The V3–V4 hypervariable regions of the 16S rRNA gene were amplified using primers 338（5′-ACTCCTACGGGAGGCAGCAG-3' ) and 806R (5′-GGACTACNNGGGTATCTAAT-3′). Negative controls without template DNA were included in each batch; only batches in which the negative control showed no detectable amplification (<0.1 ng/μL) were considered valid, thereby excluding potential aerosol contamination.

PCR amplification was performed as follows: barcoded primers targeting the V3–V4 regions were used at a concentration of 1 μM. Amplification was conducted using Phusion® High-Fidelity PCR Master Mix with GC Buffer (New England Biolabs, Cat# M0530S) to ensure high accuracy and efficiency. The PCR reaction mixture (30 μL) consisted of 10 μL template DNA, 15 μL Phusion® Master Mix, 1 μL each of forward and reverse primers (1 μM, containing Illumina adapters and sample-specific barcodes), and 2 μL nuclease-free water. The thermal cycling conditions were as follows: initial denaturation at 98 °C for 1 min; 30 cycles of denaturation at 98 °C for 10 s, annealing at 50 °C for 30 s, and extension at 72 °C for 30 s; followed by a final extension at 72 °C for 10 min and hold at 10 °C. Amplification was performed using a Bio-Rad T100 thermal cycler.

(3) PCR product purification and pooling

PCR products were pooled at equimolar concentrations and thoroughly mixed. The pooled amplicons were purified by 2% agarose gel electrophoresis (1× TAE buffer), and the target bands were excised and recovered using a Universal DNA purification kit. Fragment quality was verified using an Agilent 5400 Fragment Analyzer.

(4) Library preparation and sequencing

Sequencing libraries were constructed using the NEBNext® Ultra DNA Library Prep Kit. Library quality was assessed using the Agilent 5400 system and quantified by qPCR. Sequencing was performed on the Illumina NovaSeq 6000 platform using a paired-end 250 bp (PE250) strategy, with a minimum sequencing depth of ≥50,000 reads per sample.

(5) Bioinformatics analysis

The raw high-throughput sequencing data were processed using QIIME2 (version 2024.2). The DADA2 algorithm was employed for quality control, trimming, denoising, and assembly of the raw sequences to remove phiX, chimeric, and erroneous reads, thereby generating amplicon sequence variants (ASVs). Taxonomy was assigned to ASVs using the classify-sklearn method against the Silva 138 database ( https://www.arb-silva.de/ ).

(6) Microbial diversity

Alpha diversity: Alpha diversity of the microbial community was assessed through richness (observed ASVs and Chao1 index), diversity (Shannon and Simpson indices), evenness (Pielou’s evenness index), and coverage (Good’s coverage). Kruskal–Wallis rank sum tests were used to compare alpha diversity indices among groups. Rarefaction curves were generated by rarefying the ASV table to a series of sequencing depths and calculating the number of observed ASVs at each depth; curves approaching a plateau indicate that the sequencing depth was sufficient to capture the majority of microbial diversity.

Beta diversity: Beta diversity, representing between-sample differences in community composition, was evaluated by computing Bray–Curtis distance and Jaccard distance based on ASV abundances. Statistical significance of group differences was determined by permutational multivariate analysis of variance (PERMANOVA) using the adonis function. Nonmetric multidimensional scaling (NMDS) and principal coordinate analysis (PCoA) were performed based on the distance matrices and visualized using the vegan and ggplot2 packages in R.

Biomarker discovery: Between-group differences in microbial community structure were assessed using permutational multivariate analysis of variance (PERMANOVA), a non-parametric statistical test. Pairwise comparisons were performed as post-hoc tests, with p-values adjusted using the false discovery rate (FDR) method.

Keystoneness calculation:Structural Keystoneness (Ks) was calculated using the deep learning-based keystone species identification framework proposed by Wang et al^[^^[1]]^.This method constructs a neural network model from species abundance data and assesses the structural importance of each taxon by sequentially removing it and quantifying the resulting reduction in community prediction accuracy, thereby assigning a keystone-ness score to each taxon. Specifically, a trained deep learning model is used to predict the original intact community; the abundance of the target species is then set to zero and the community is re-predicted. The difference in prediction error between the two conditions represents the raw keystone-ness score of that species. Based on these scores, the median keystone-ness [median(Ks)] and the median absolute deviation of keystone-ness [MAD(Ks)] across all samples were calculated for each taxon, to evaluate the community specificity and condition dependence of its ecological function.

**Supplementary method 2: Three-Day Dietary Assessment**

Prior to fecal sample collection, all participants completed a 3-day dietary assessment (two weekdays and one weekend day) to characterize their habitual dietary intake before stool sampling. A 3-day food record is a widely accepted method for assessing short-term habitual dietary intake and has been extensively used in nutritional epidemiology and gut microbiota research **(Thompson & Subar, 2017; Shim et al., 2014)**.

Participants were instructed to maintain their usual dietary habits throughout the recording period and were not provided with identical meals or nutritionally standardized diets. They were asked to record all foods and beverages consumed over three consecutive days, including meal times, food items, estimated portion sizes, cooking methods, snacks, and beverages. To improve the completeness and accuracy of the dietary records, participants received detailed verbal instructions from trained investigators before recording, and all completed dietary records were subsequently reviewed. Any ambiguous or incomplete entries were clarified through follow-up interviews.

Dietary intake was analyzed using the China Food Composition Table (6th Edition) to estimate daily energy intake, carbohydrate, protein, fat, and dietary fiber intake **(Yang et al., 2018)**. These dietary variables were subsequently compared among the three physical activity groups to evaluate potential differences in habitual dietary intake.

Because no identical or nutritionally standardized meals were provided before fecal sample collection, residual dietary variation among participants cannot be completely excluded and should be considered when interpreting the findings.

**References：**

Thompson FE, Subar AF. Dietary Assessment Methodology**.** In: Coulston AM, Boushey CJ, Ferruzzi MG, Delahanty LM, editors. *Nutrition in the Prevention and Treatment of Disease*. 4th ed. Academic Press; 2017. p. 5–48.

Shim JS, Oh K, Kim HC. Dietary assessment methods in epidemiologic studies. Epidemiology and Health. 2014: e2014009.

Yang YX, Wang GY, Pan XC. China Food Composition Tables (Standard Edition). 6th ed. Beijing: Peking University Medical Press; 2018.

**Supplementary Figures1:** Comparison of the relative abundance of different species

**Supplementary Tables2:** Complete list of Ks value rankings for each genus in species key analysiss

| Species name | Median (Ks) |
| --- | --- |
| g__Ruminococcus | 0.043463060907749 |
| g__Faecalibacterium | 0.037329837740803 |
| g__Dialister | 0.037052204230984 |
| g__norank_f__[Eubacterium]_coprostanoligenes_group | 0.034121664972367 |
| g__Phascolarctobacterium | 0.025535140227432 |
| g__Bifidobacterium | 0.02330653617453 |
| g__Erysipelotrichaceae_UCG-003 | 0.022969560104002 |
| g__Collinsella | 0.021141744072199 |
| g__unclassified_f__Enterobacteriaceae | 0.021069106447491 |
| g__Bacteroides | 0.020952658061171 |

1. ^[]^ Wang XW, Sun Z, Jia H, Michel-Mata S, Angulo MT, Dai L, He X, Weiss ST, Liu YY. Identifying keystone species in microbial communities using deep learning. *Nat Ecol Evol*. 2024;8(1):22-31.
